# Symptoms of central sensitization and comorbidity for juvenile fibromyalgia in childhood migraine: an observational study in a tertiary headache center

**DOI:** 10.1186/s10194-017-0764-8

**Published:** 2017-05-30

**Authors:** Marina de Tommaso, Vittorio Sciruicchio, Marianna Delussi, Eleonora Vecchio, Marvita Goffredo, Michele Simeone, Maria Grazia Foschino Barbaro

**Affiliations:** 1 Applied Neurophysiology and Pain Unit, Basic Medical, Neuroscience and Sensory System Department, Bari Aldo Moro University, Giovanni XXIII Building, Policlinico General Hospital, Via Amendola 207, A 70124 Bari, Italy; 2 Children Epilepsy and EEG Center, Triggiano, Bari, Italy; 3 Psychological Pediatric Service, Bari Policlinico General Hospital, Bari, Italy

**Keywords:** Migraine, Children, Allodynia, Pericranial tenderness, Juvenile fibromialgia

## Abstract

**Background:**

Central sensitization is an important epiphenomenon of the adult migraine, clinically expressed by allodynia, pericranial tenderness and comorbidity for fibromyalgia in a relevant number of patients. This study aimed to evaluate the frequency and the clinical characteristics of allodynia, pericranial tenderness, and comorbidity for Juvenile Fibromialgia (JFM) in a cohort of migraine children selected in a tertiary headache center.

**Methods:**

This was an observational cross-sectional study on 8–15 years old migraine patients. Allodynia was assessed by a questionnaire. Pericranial tenderness and comorbidity for JFM as well as their possible association with poor quality of life and migraine related disability, and with other clinical symptoms as anxiety, depression, sleep disorders and pain catastrophizing, were also evaluated.

**Results:**

One hundred and fifty one patients were selected, including chronic migraine (n°47), migraine without aura (n° 92) and migraine with aura (n° 12) sufferers. Allodynia was reported in the 96,6% and pericranial tenderness was observed in the 68.8% of patients. Pericranial tenderness was more severe in patients with more frequent migraine and shorter sleep duration. Allodynia seemed associated with anxiety, pain catastrophizing and high disability scores. Comorbidity for JFM was present in the 0.03% ofpatients. These children presented with a severe depression and a significant reduction of quality of life as compared to the other patients.

**Conclusions:**

This study outlined a relevant presence of symptoms of central sensitization among children with migraine. Severe allodynia and comorbidity for JFM seemed to cause a general decline of quality of life, which would suggest the opportunity of a routine assessment of these clinical features.

## Background

Migraine is a chronic neurological disorder, with an early onset in childhood and juvenile age. Its genetic basis, though multifactorial, is confirmed by its occurrence in children with first degree inheritance for the disorder [1, 2]. The clinical picture of juvenile headache is different from the adult form, and in fact the last International Classification of Headache Disorders, 3rd beta edition, includes footnotes for migraine diagnostic criteria in childhood [3, 4]. Central sensitization is an important epiphenomenon of the migraine attack. It is caused by functional modifications of the trigeminal and cervical nociceptive systems and may favor headache worsening and persisting [2]. Allodynia is the clinical manifestation of the central sensitization [5]. It was described in almost the 70% of episodic migraine patients and in the 90% of chronic migraine sufferers, as a factor predicting the chronic evolution of the disease [6, 7]. In a recent study performed in a large cohort of primary headache patients, allodynia was more represented in the group of chronic migraine patients, with a positive correlation with anxiety, depression and disability [8]. In the same study, a short sleep duration seemed to facilitate the symptoms of central sensitization in adult primary headache [8]. In clinical practice, allodynia is measured by a questionnaire validated in large samples of patients in adult age, affected by migraine and other primary headaches [9, 10]. Pericranial tenderness [11] is a sign of persistent activation of the trigeminal and cervical nociceptors, which is present also in the asymptomatic phase of patients with frequent and chronic migraine. [12]. Recent studies reported the presence of symptoms of central sensitization even in childhood migraine [13, 14]. In those studies, allodynia was retrospectively assessed by the use of simplified questionnaires, which allowed to ascertain that this symptom was present in a large number of migraine children [14]. Other signs of central sensitization, as pericranial tenderness or diffusion of pain symptoms outside the trigemino-cervical districts, as for fibromyalgia comorbidity, were rarely assessed in childhood migraine [15, 16] In addition, children with migraine are frequently characterized by psychopathological traits which may facilitate the chronic evolution [17], though their possible influence on central sensitization symptoms may evolve differently than in adult migraine. In a recent study on pain-related evoked potentials in children with migraine, we observed reduced habituation of the cortical responses to pain, which was correlated with allodynia [16]. This latter symptom was found in almost all patients, suggesting a higher representation in the juvenile migraine than in the adult form [16].

Fibromyalgia (FM) is an invalidating painful disorder, whose different aspects may be explained by the increased central sensitization phenomena [18]. A growing number of studies are dealing with Juvenile Fibromyalgia (JFM), which is emerging as a well-documented phenomenon in youth, with studies showing prevalence rates ranging from 2 to 6% of school-age children [19–21]. The criteria of diagnosis are similar to those for adult Fibromyalgia [22, 23]. Taking into consideration the impact of FM on the global burden of adult migraine [24, 25], the assessment of frequency and clinical profile of patients with JFM comorbidity would be useful to recognize this potentially disabling aspect of migraine.

The present study aimed to.

1) evaluate the frequency of allodynia, pericranial tenderness and JFM in a population of children with migraine selected in a tertiary headache center.

2) assess if the severity of allodynia,pericranial tenderness and fibromyalgia might be associated a) with the headache-related disability and the interference of pain on quality of life b) with other clinical features as anxiety, depression, pain catastrophizing and sleep disturbance.

## Methods

**Fig. 1:**
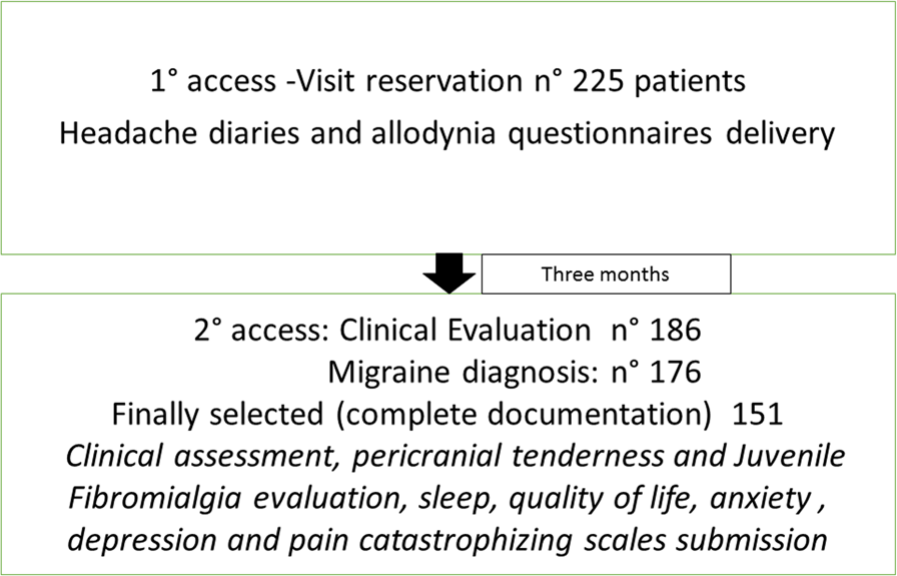
Flow chart reporting main study design

Subjects-This was a cross-sectional observational study on childhood migraine. The 8- to 15-years-old children and adolescents were selected among 2400 outpatients suffering from headache, examined consecutively at the Neurophysiopathology of Pain Unit of Bari University between January 2013 and June 2015. This is a tertiary center for headache and chronic pain that is dedicated to both adults and children on distinct days. The inclusion criteria were a diagnosis of migraine without aura (MA cod. 1.1), migraine with aura (MWA cod 1.2.1) and chronic migraine (CM 1.3), according to the International Classification of Headache Disorders [3]. The exclusion criteria for the present evaluation were the untaking of actual preventive treatments and/or any central nervous system acting drug therapy, comorbidity with general medical diseases and with any other neurologic condition. The diagnosis of migraine was confirmed on the basis of at least 3 months of observation preceding the first visit. Upon the first visit reservation, the hospital staff gave parents the headache diary and the questionnaire of allodynia in the adult version [10, 16], to be presented during the first clinical approach at our Center (Fig. 1).

### Clinical assessment

All migraine patients and their parents were carefully interviewed by the neurologists and psychologists staff on their first visit to our center. The frequency of the headache was evaluated through the diaries where patients and their parents had reported the days with headache in the last 3 months. Then patients were divided into four categories of frequencies (1–4; 5–9; 10–14; 15–30 days/month).

### Pericranial tenderness

It was evaluated in all cases, in accord with previous studies performed in adult and young patients with headache [8, 11, 16].

### Allodynia

Mothers were requested to interview children during migraine attack, in order to help them in filling the allodynia questionnaire for each migraine attack. We proposed the same questionnaires employed for adults [8, 16], consisting of the symptoms checklist reported by Lipton et al. [10]. For the allodynia severity, the average number of allodynia symptoms across different attacks was considered. The allodynia questionnaire is not presently adapted for children, so we suggested the parents to indicate as “not applicable” those questions specific to adults. We classified patients as allodynic based on the presence of at least one symptom reported in the questionnaire in over the 50% of the headache episodes.

### Juvenile Fibromyalgia comorbidity

In order to individuate the migraine children with the comorbidity for JFM, we applied the most recent criteria for adult Fibromyalgia [22], recently validated also in adolescents [23]. Symptoms of fibromyalgia were examined using the Widespread Pain Index (WPI) and Symptom Severity Index (SSI). These measures were adapted from the 2010 ACR diagnostic criteria for FM [22] and designed to gather information on fibromyalgia symptoms through the children and parents reports.

### Migraine disability

The childhood version of the Migraine Disability Assessment was used (PedMIDAS) [26].

Quality of life assessment-The Italian version of the Pediatric Quality of Life Inventory (PedsQL) is one of the most used quality of life measures for children aged 2–18 years. Specifically, the child self-report (PedsQL) includes the 5–7 years old younger children, the 8–12 aged children, and the 13–18 years old teens, the second and the third items employed in the present study. The parent proxy report (PedParQL) also includes ages 2–4 (toddlers), 5–7 (younger children), 8–12 (children), and 13–18 (teens). The two forms, for children and parents, are parallel, providing an indication of the child’s and the parent’s perception. The instrument instructions ask how frequently a problem was present during the past 1 month. In the present study, we considered the PedsQL and PedParQL total scores, Physical functioning (PedsQLP, PedParQLP) and Emotional functioning (PedsQLE, PedParQLE) sub scores [27].

### Psychopathological assessment

The Psychiatric Self-Administration Scales for Youths and Adolescents was used. [28, 29]. This is an Italian standardized battery which includes self-report scales for the assessment of a wide range of psychiatric symptoms according to the DSM V diagnostic criteria.

The entire battery includes six scales (each with subscales), which can be used together or separately, all provided with satisfactory psychometric properties. The anxiety (SAFA A) and depression (SAFA D) scales were employed for the purposes of the present study.

### Sleep assessment

The SDSC (Sleep Disturbance Scale for Children) questionnaire was used to assess quality and disturbances of sleep over the past 6 months in our young patients [30]. In this study the total SDSC score (SDSC-T), and the sub-score SDSC-tt for the total time of sleep were considered. This latter item includes a 1–3 scale to grade total sleep duration. For both the indexes higher values correspond to worse sleep performance.

### Pain Catastrophizing

The Pain Catastrophizing Scale for Children [31] is the child version of the Pain Catastrophizing Scale developed by Sullivan et al. [32]. Sullivan et al. [32] characterized pain catastrophizing as an “exaggerated negative ‘mental set’ brought to bear during actual or anticipated pain experience”. This 13-item self-report instrument assesses the tendency to catastrophize about pain and three dimensions of catastrophizing, rumination, magnification and helplessness that are subsumed under the higher-order construct of pain catastrophizing. Furthermore, according to the authors, [31] the total Pain Catastrophizing score (PCS-S) predicts pain severity and pain-related disability in pediatric pain patients.

The study was approved by the local Ethic Committee of Bari Policlinico General Hospital. Parents and children were informed about the study and parents signed an informed consent, regarding the possibility that the clinical data would be used in anonymous form for scientific purposes, while the entire database would be visible only to the local neurologists’ staff, without permission for public access.

### Statistical analysis

Analyses were conducted using IBM SPSS Statistics for Windows, Version 21.0 (IBM Corp., 2011, Armonk, NY: http://www.spss.com). Demographic data, headache frequency, headache-related disability, were summarized using means and standard deviation, taking into consideration migraine subgroups. The allodynia and pericranial tenderness were compared among migraine groups by means of the Chi-square test and ANOVA test. Separate linear regression models were used to evaluate if allodynia and pericranial tenderness total score were correlated to headache-related disability and quality of life, and in a separate analysis to other clinical features as anxiety, depression, pain catastrophizing and sleep disorders, with the frequency of migraine as covariate. A value of alpha 0.008 was used for the regression analysis to adjust for multiple comparisons. All tests were two-tailed.

To evaluate the effect of JFM comorbidity on disability indices and other clinical variables as anxiety, depression, pain catastrophizing and sleep disorders, a MANOVA analysis with JFM comorbidity as factor was initially designed. Considering the low number of JFM patients, the clinical features of patients with JFM were summarized using mean and standard deviations and compared with those of patients without fibromyalgia by means of one-way ANOVA analysis.

## Results

Among 225 children with headache who approached our Center, 20 received a diagnosis of probable migraine, 25 was diagnosed as tension type headache patients, 25 did not complete the diaries of headache and or/allodynia questionnaires and four were lost to the first visit. We finally selected 151 migraine patients (Fig. 1; Table 1).

**Table 1:** Mean (M) and Standard Deviations (SD) of demographic and clinical features of migraine patients

	Sex		Age (years)	Migraine onset (months)	Frequency of headache (days/migraine /month)	PedMIDAS
MIGRAINE WITHOUT AURA (MA) Cod 1.1 (43 males. 49 females)	males 43 females 49		M 10.87 SD 2.91 (10.3–11.5)	M 12.2 SD 3.4 (4–17)	M 6.7 SD 2.3 (3–13)	M 12.64 SD 12.92 (0.8–32.2)
CHRONIC MIGRAINE (CM) Cod 1.3 (20 males. 27 females)	males 20 females 27		M 10.98 SD 2.96 (10.1–11.8)	M 10.2 SD 6.1 (5–15)	M 17.7 SD 2.5 (16–30)	M 23 SD 21 (17–30.1)
MIGRAINE WITH AURA (MWA) Cod. 1.2.1 (3 males. 9 females)	males 3 females 9		M 11.75 SD 3.11 (9.8–13.5)	M 11.2 SD 4.5 (6–14)	M 2.3 SD 2.1 (1–6)	M 16.25 SD 27.47 (0.8–30)
Chi square p	2.07 n.s.	ANOVA P Bonferroni	F 0.47 n.s.	F 1.23 n.s.	F = 5.29 0.006 CM vs MWA – MA *p* < 0.01	F = 6.13 0.003 CM vs MA and MWA *p* < 0.05

*PedMIDAS* Pediatric Migraine Disability Scale

According to the headache diagnosis (class). In the first columns. The results of the chi square test are reported. in the remainder. The ANOVA test with group as factor and post-hoc Bonferroni test are showed. The 95% Confidence Intervals are reported in brackets

Demographic and clinical characteristics of migraine patients. Patients with Chronic Migraine (CM), Migraine without Aura (MA) and Migraine with Aura (MWA) were similar in age. In the three groups, females prevailed though not significantly. (chi square 2.07, n.s.) (Table 1). The frequency of headache was similar between MWA and MA groups, though slightly higher in the latter group, and obviously increased in the CM group (Table 1). Migraine disability prevailed in chronic migraine children, as compared to the other groups (Table 1).

**Fig. 2:**
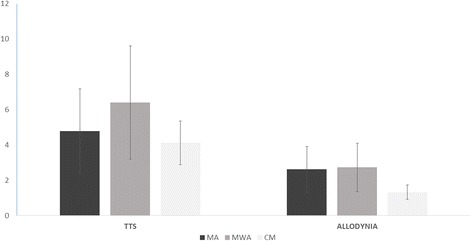
Mean and Standard Deviations of symptoms of central sensitization (TTS, Total Tenderness Score) in migraine children. Divided Into headache diagnosis groups (MA Migraine Without Aura, MWA Migraine With Aura, CM Chronic Migraine. For TTS ANOVA test with group as factor: F 4.09, *p* < 0.0009; for allodynia F 0.95 n.s. The results of Bonferroni test are reported: CM vs MA and MWA * *p* < 0.05

Symptoms of central sensitization in migraine groups- Pericranial tenderness-The most of migraine children presented with signs of pericranial tenderness. In fact, it was present on average in the 68.38% of migraine patients (66 MA, 30 CM and 10 MWA, chi square 1.43, n.s.) . The intensity of muscle pain was significantly increased in the CM group (Fig. 2). The TTS score was not associated to the disability of migraine and to the quality of life. (Table 2 a), but was significantly related to reduced sleep duration (Table 2 b).

**Table 2:** Linear regression analysis between Total Tenderness Score (TTS) score and a) pediatric Migraine Disability (PedMIDAS),Total Pediatric Quality of life-Patient (PedQL-T), Physical Pediatric Quality of life-Patient (Ped-QLPHY), Psychic Pediatric Quality of life-patient (Ped-QLQPSY), Total Pediatric Quality of life-Parent (PedPa QL-T), Physical Pediatric Quality of life-Parent (PedPar-QLPHY), Psychic Pediatric Quality of life-parent (PedPar-QLQPSY) b) total Self-Administration Scales for Youths and Adolescents-Anxiety (SAFAA-T), Total Self-Administration Scales for Youths and Adolescents-Depression (SAFAD-T), SDSC (Sleep Disturbance Scale for Children), total time of sleep (SDSC-tt), Total Pain Catastrophizing (PCS-S). (df: degree of freedom)

a						
R2	Standard deviation estimation error	Sum of squares	df	F	Sig.	
0.06	17.36	Regression 2316.96	7	1.10	0.211	
		Residual 37,653.81	143			
		Total 39,970.77	150			
Model			t	Sig.	95% CI	
		Beta			Low	High
	PedMIDAS	−0.11	−1.12	0.26	−0.06	0.02
	PQL_ado_TOT	0.59	1.79	0.08	−0.03	0.52
	PQL_ado_Fis	−0.24	−1.29	0.20	−0.21	0.04
	PQL_ado_PsSc	−0.49	−2.06	0.04	−0.33	−0.01
	PQL_gen_TOT	−0.03	−0.10	0.92	−0.25	0.23
	PQL_gen_Fis	0.04	0.16	0.88	−0.13	0.15
	PQL_gen_PsSc	−0.01	−0.05	0.96	−0.14	0.13
b						
R2	Standard deviation Estimation Error	df	Sum of squares		F	Sig.
0.02	16.67	Regression 5	380.74		1.37	0.11
		Reisidual 145.00	277.98			
		Total 150.00				
Model						
Beta		T	Sig.		95% CI	
					Low	High
SAFA_A-TOT	−0.02	−0.12	0.91		−0.15	0.13
SAFAD_TOT	−0.06	−0.43	0.67		−0.18	0.11
SDSC_TOT	−0.06	−0.68	0.50		−0.16	0.08
SDSC_tt	0.18	2.1	0.048		0.00	2.28
PCS-S	0.15	1.53	0.13		−0.02	0.14

Allodynia- Among the 12 symptoms reported in the allodynia questionnaires for adult patients, according to Lipton et al. [10] the items which were considered available by mothers varied from 6 to 11 (7.65 + 2). The items of 100% use were to comb your hair, to wear tight clothing, taking a shower, resting your face or head on the pillow, exposure to heat, exposure to cold.

Almost all patients reported allodynia (146 among 151). The prevalence of allodynic patients was relevant in all migraine groups, though in reduced proportion in MWA patients (90 MA patients, 45 CM, 10 MWA, chi-square 9.022 p 0.001). The severity of allodynia was similar among groups, considering both diagnosis and frequency of migraine (Fig. 2). Linear regression analysis showed a significant relationship between allodynia severity and the sum of the scores of quality of life and headache disability, despite no single score reached a statistical significant association. (Table 3 a). The total of clinical symptoms, as anxiety, depression, sleep quality and quantity and pain catastrophizing, showed to be significantly related to allodynia severity. Higher anxiety and pain catastrophizing scores were significantly associated to more severe allodynia (Table 3 b).

**Table 3:** Linear regression analysis between number of allodynia symptoms and a) pediatric Migraine Disability (PedMIDAS),Total Pediatric Quality of life-Patient (PedQL-T), Physical Pediatric Quality of life-Patient (Ped-QLPHY), Psychic Pediatric Quality of life-patient (Ped-QLQPSY), Total Pediatric Quality of life-Parent (PedPa QL-T), Physical Pediatric Quality of life-Parent (PedPar-QLPHY), Psychic Pediatric Quality of life-parent (PedPar-QLQPSY) b) total Self-Administration Scales for Youths and Adolescents-Anxiety (SAFAA-T), Total Self-Administration Scales for Youths and Adolescents-Depression (SAFAD-T), SDSC (Sleep Disturbance Scale for Children), total time of sleep (SDSC-tt), Total Pain Catastrophizing (PCS-S). (df: degree of freedom)

a						
R2	Standard deviation estimation error	Sum of squares	df	F	Sig.	
0.10	5.56	Regression 675.472	7.00	3.12	0.004	
		Residual 4149.138	143			
		Total 4824.609	150			
Model			t	Sig.	95% CI	
		Beta			Low	High
	PedMIDAS	−0.07	−0.78	0.43	−0.02	0.01
	PQL_ado_TOT	−0.56	−1.85	0.07	−0.17	0.01
	PQL_ado_Fis	−0.05	−0.31	0.76	−0.05	0.03
	PQL_ado_PsSc	0.40	1.79	0.08	0.00	0.10
	PQL_gen_TOT	0.19	0.57	0.57	−0.05	0.10
	PQL_gen_Fis	−0.22	−1.01	0.31	−0.06	0.02
	PQL_gen_PsSc	−0.13	−0.68	0.50	−0.06	0.03
b						
0.09	5.71	Regression 589.37	5	3.61	0.004	
		Residual 4082.23	145			
		Total 4671.60	150			
Model			t	Sig.	95% CI	
		Beta			Low	High
	SAFAA_ T	0.50	3.70	0.001	0.04	0.14
	SAFAD_T	−0.35	1.29	0.20	−0.11	−0.01
	SDSC	−0.03	−0.31	0.75	−0.04	0.03
	SDC-tt	0.07	0.80	0.43	−0.23	0.53
	PCS-S	0.11	2.59	0.01	−0.01	0.04

**Fig. 3:**
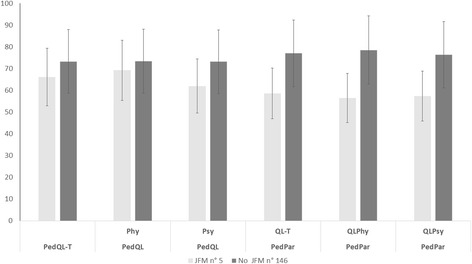
Quality of life In Juvenile Fibromyalgia (JFM) patients. Total Pediatric Quality of life-Patient (PedQL-T). Physical Pediatric Quality of life-Patient (Ped-QLPHY) . Psychic Pediatric Quality of life-patient (Ped-QLQPSY). Total Pediatric Quality of life-Parent (PedPa QL-T). Physical Pediatric Quality of life-Parent (PedPar-QLPHY) . Psychic Pediatric Quality of life-parent (PedPar-QLQPSY). ANOVA test with group as factor: PedQL-T, F 4.39, p 0.014; Ped-QLPHY, F 3.76, p 0.026; Ped-QLQPSY, F 3,66, p 0.028; PedPa QL-T, F 4.74, p 0.01; PedPar-QLPHY, F 4.47 p 0.013; PedPar-QLQPSY F 3.33, p 0.038

Comorbidity for Juvenile Fibromyalgia Five migraine patients fulfilled the criteria for JFM (0.03%). They were similar in age to the remainder of patients, but presented with higher allodynia scores, more severe pericranial tenderness, higher depression score, while sleep disturbances and pain catastrophizing were not significantly increased in FM patients. (Table 4). Patients with JFM presented with poor quality of life, for both physical and psychical functions, as compared to the other migraine patients (Fig. 3).

**Table 4:** Juvenile Fibromyalgia (JFM) comorbidity in young migraine

	Age	Frequency	Ped MIDAS	TTS	ALL	SAFAA-T	SAFAA-D	SDSC	SDSC-tt	PCS-S
JFM 3 M 2 F	M 10.9 SD 2.9 (9.9–15)	M 18.1 SD 4.5 (15–29)	M 28.6 SD 18 (5–40)	M 11.8 SD 4.3 (4–15)	M 3.2 SD 1.3 (2.2–5.9)	M 59.8 SD 7.3 (47–73)	M 57.4 SD14.4 (49–70)	M 67.2 SD 17.6 (38–56)	M 2.3 SD 0.5 (1.3–3)	M 20.1 SD 17.7 (5–32)
No JFM 63 M 83 F	M11 SD3.3 (10.7–13.3)	M 6.5 SD 5.6 (2–14)	M 18.8 SD 20.1 (12–20)	M 4.9 SD 4.9 (4–6)	M 2.5 SD 1.6 (2.2–2.8)	M 55.1 SD 11.6 (52–57)	M 52.4 SD 9.9 (50–53)	M 55.8 SD 11.4 (37–40)	M 2.1 SD 0.8 (2–2.3)	M 17.4 SD 11.1 (18–23)
ANOVA F P	0.18 n.s.	8.56 0.0003	F 6.22 0.014	F = 5.72 0.004	F = 3.36 0.037	F = 2.35 n.s.	F = 8.49 0.0003	F = 2.31 n.s.	F = 2.32 n.s.	F = 1.81 n.s.

Mean (M) and Standard Deviations (SD) of demographic and clinical features are reported. The 95% Confidence Intervals are indicated in bracket. Frequency of headache considered the average number of days with headache in a month. Computed over 3 months. Pediatric Migraine Disability (PedMIDAS). Total Tenderness score (TTS). number of allodynia symptoms (ALL). Total Self-Administration Scales for Youths and Adolescents-Anxiety (SAFAA-Tot). Total Self-Administration Scales for Youths and Adolescents-Depression (SAFAD-T). Sleep Disturbance Scale for Children (SDSC). total time of sleep (SDSC-tt). Total Pain Catastrophizing (PCS-S) are shown. The ANOVA test with group as factor is presented

## Discussion

The present results confirmed high frequency of pericranial tenderness and allodynia in a population of children with migraine selected in a tertiary headache center. While pericranial tenderness was prevalently present in the chronic migraine, allodynia was present also in children with episodic headaches, associated with the globality of disability scores, anxiety and pain catastrophizing. Few children presented with comorbidity for JFM, though these patients were characterized by a very disabling disease. In the next paragraphs, the single results are commented.

### Clinical characteristics of migraine

We found a large group of young patients with CM among the included cases (31, 1%). This group seemed larger if compared to studies conducted in adults with chronic migraine, recruited in the same center [8]. In the general population, the prevalence of CM in children and adolescent could be regarded as roughly 1.5–1.8%, not dissimilar from the adult population, though with a tendency to increase after the 12 years [33]. We can suppose that in childhood age, the approach to the tertiary Headache Centers is specially requested for severe migraine, a hypothesis that may be confirmed by the high scores of migraine disability reported by these patients. These data seem worthy of confirmation in larger samples. Another observation, which may emerge from these data, regards the equilibrium between sexes in the observed population, which confirms the results of epidemiological studies in prepubertal children [34–36]. As expected, the disability linked with migraine prevailed in the CM group. In general, in patients with recurrent migraine, 8 days are lost at school because of headache, where the most bothersome features seem to be the intensity and the duration of the headache. For these reasons, school absence is expected to be prominent in the CM patients [37].

### Symptoms of central sensitization in migraine and correlation with clinical and psychopathological features –Pericranial tenderness

The most of migraine patients presented pericranial tenderness, independently of the type of migraine, in accord with previous studies [15]. The intensity of pericranial muscle pain was significantly higher in patients with chronic migraine as compared to those with episodic migraine. The comparison among the different groups of headache frequency, showed also a significant reduction in patients with rare attacks. Pericranial tenderness may be caused by hyperalgesia, for the persistence of central sensitization between the migraine attacks. The close relation between an increase in pain perception and chronic headache, observed in adults with primary headache forms [12], may be confirmed also in children migraine. The correlation between pericranial tenderness and short sleep duration, confirmed what we previously observed in adults, that the loss of sleep could facilitate central sensitization [8]. Children who suffer from headache have usually a high rate of sleep difficulties, including insufficient sleep time, frequently caused by nocturnal migraine onset [38, 39]. The presence of a self-sustained circuit of sleep reduction, central sensitization and possible evolution into chronic migraine [8], might be supposed even in juvenile age. The linear regression analysis, where the effect of the headache frequency was subtracted, indicated that pericranial tenderness is not an independent factor of disability; rather it may be a consequence of the chronic headache.

#### Allodynia

In the most of migraine attacks, children presented allodynia. Its prevalence among migraine patients was even higher than that reported in previous studies, which were based on a retrospective evaluation by a simplified questionnaire [14], very similar to that used in the present study. The prevalence of allodynia in adults approaches the 70% in large migraine populations, selected by mailing questionnaires [10]. In our previous study in a large population of adults with primary headache forms, in whom allodynia was evaluated on the basis of 3 months observation, its frequency reached the 91% [8], so the longitudinal observation may be more sensitive than the retrospective assessment in detecting symptoms associated to migraine attack. The six items selected by children to describe allodynia during acute migraine, are similar to those proposed by Raieli et al. [14]. The questionnaire [9, 10] was easily and diligently compiled with the support of parents, based on children symptoms, so it might be proposed in the routine assessment of childhood migraine. The presence of allodynia seemed reduced in patients with migraine with aura, though this was a too small group to be considered for conclusions. A sample enlargement seems thus mandatory in order to confirm these data. In fact, in adult patients reporting aura symptoms, allodynia is more frequent than in those without aura [10], so, in our opinion, this finding is an effect of the reduced number of patients rather than be a feature of juvenile migraine with aura. Allodynia may be confirmed as a common symptom in childhood migraine, present in the most of episodic and chronic patients. The average number of reported allodynia symptoms appeared similar to that described in adult migraine [8], though it was computed on a reduced number of available items. In adults with primary headache forms, the number of allodynia symptoms seemed similar between episodic and chronic migraine [8], a finding that may be confirmed also in children. The utility of allodynia questionnaire in the routine assessment of children with migraine may be also suggested by its association with the disability scores. In fact, despite we did not find any significant association between allodynia and the single scores of the pediatric MIDAS or the quality of life scale, the globality of these tests appeared to be significantly correlated with the allodynia severity. Allodynia severity, in terms of symptoms number and intensity, appeared to be similar among patients with chronic and episodic migraine, at least in those patients with the form without aura. This might suggest that allodynia would be a cause of disability of each single migraine attack, worsening its evolution and hampering child’s ability in daily activities. The number of allodynia symptoms reported on average in the migraine attacks, appeared significantly correlated to anxiety, which would enhance the sensitivity to pain and would reduce the threshold of central sensitization phenomenon. In a recent study performed in a large cohort of adults suffering from primary headaches [8], allodynia was correlated with anxiety and depression in patients with migraine and tenson type headaches, both chronic and episodic. There is evidence that psychopathological factors may facilitate the development of allodynia for the failure of the descendant control for to pain modulation [40]. Child migraine is frequently associated with anxiety and depression [41]. However, anxiety, more than depression, would be predictive of the long-term migraine persistence and the headache-related disability [42–44], for a probable linkage to the phenomena which might facilitate central sensitization. Pain catastrophizing [31] appeared relevant in patients with severe allodynia, as might be expected for its association with high pain perception… Catastrophizing of pain was rarely assessed in children migraine, but its possible role in the facilitation of central sensitization [45, 46] could indicate it as a useful parameter for clinical assessment [47]. The globality of the scores referring to anxiety, depression, pain catastrophizing and sleep was also associated to severe allodynia, though an enlargement of cases series might probable enable significant correlations with the single scores.

#### Juvenile fibromyalgia comorbidity in migraine children

Few children among our migraine population presented comorbidity for JFM. At the best of our knowledge, this is the first study assessing this comorbidity in children with migraine and its low frequency in respect to adult migraine [2, 8, 24, 25]. Juvenile Fibromyalgia is currently emerging as an invalidating disease, even in developmental age [19–21]. Although it appears a less common condition than in adults, chronic widespread pain is a well-documented phenomenon in youth, with studies showing prevalence rates ranging from two to 6% of school-age children [48]. The five children affected by JFM, showed a clinical picture of particular severity, with relevant psychopathological traits, especially depression, remarkable presence of pericranial tenderness and allodynia, and further reduction of quality of life as compared to the other migraine children. In accord with adult populations [8, 24, 25], also the patients with JFM suffered from chronic migraine, confirming that a general facilitation of central sensitization could explain a clinical phenotype characterized by chronic headache and diffuse skeletal pain [2]. Anxiety, sleep disturbances and pain catastrophizing, seemed not to prevail in this migraine subgroup, though these aspects would deserve further assessment in larger cohorts of .migraine patients. In a longitudinal study of adolescents with JFM, followed till the early adulthood, it was found that approximately in the 20th year of age, lifetime prevalence of comorbidities, including anxiety and mood disorders, approached that found in adult studies, with over 75% of cases with an anxiety disorder and at least one episode of major mood disorder (typically major depressive disorder) [49]. More cases are needed in a next future to define the impact of this comorbidity on the global burden of childhood headache. Vegetative dysfunction, joint laxity or hypermobility, sleep disorders and psychiatric tracts, are commonly described in children with JFM [21], while headache was not previously evaluated as a frequently associated condition. Despite the low frequency of JFM comorbidity among children with migraine, the severe disability presented by these young patients, could indicate the opportunity to assess the possible presence of diffuse pain at least in children with chronic migraine.

### Study limitations

Despite our sample, size was quite large; the variability of clinical symptoms in developmental age would indicate the utility of group’s enlargement. An important point in studies on child’s headache is the utility of clinical follow-up, in order to confirm migraine diagnosis and the significance of symptoms of central sensitization in the natural history of the disease. Another relevant aspect to be clarified in future studies would be the frequency and influence of first degree inheritance for migraine, symptoms of central sensitization and fibromyalgia.

## Conclusions

This study outlined a relevant presence of symptoms of central sensitization among children with migraine. Patients with severe allodynia and comorbidity for JFM presented with a general decline of quality of life, which would suggest the opportunity of a routine assessment of these features, in light of a better understanding of their role in the global outcome of the disease and the response to treatments. Psychiatric comorbidity seems to have a major role in central sensitization at this age, as anxiety seems to enhance allodynia during single attacks, and depression seems to favor the development of JFM, so their careful assessment and management would be of primary importance to prevent the evolution into severe disability.

### Clinical implications

The majority of children with migraine are characterized by allodynia and pericranial tenderness.

The comorbidity for Juvenile Fibromyalgia is an infrequent but disabling condition.

The correlation between symptoms of central sensitization and poor quality of life supports the utility of their routine assessment in children with migraine.

## Abbreviations

CM: Chronic migraine
JFM: Juvenile Fibromyalgia
MA: Migraine without aura
MWA: Migraine with aura
PCS-S: Total pain catastrophizing
PedMIDAS: Pediatric migraine disability
PedPa QL-T: Total pediatric quality of life-parent
PedPar-QLPHY: Physical pediatric quality of life-parent
PedPar-QLQPSY: Psychic pediatric quality of life-parent
Ped-QLPHY: Physical pediatric quality of life-patient
Ped-QLQPSY: Psychic pediatric quality of life-patient
PedQL-T: Total pediatric quality of life-patient
SAFAA-T: Total self-administration scales for youths and adolescents-anxiety
SAFAD-T: Total self-administration scales for youths and adolescents-depression
SDSC: Sleep disturbance scale for children
SDSC-tt: Total time of sleep
TTS: Total tenderness score
